# Binder Jet 3D Printing of Compound LEV-PN Dispersible Tablets: An Innovative Approach for Fabricating Drug Systems with Multicompartmental Structures

**DOI:** 10.3390/pharmaceutics13111780

**Published:** 2021-10-25

**Authors:** Xiaoxuan Hong, Xiaolu Han, Xianfu Li, Jiale Li, Zengming Wang, Aiping Zheng

**Affiliations:** 1 State Key Laboratory of Toxicology and Medical Countermeasures, Beijing Institute of Pharmacology and Toxicology, Beijing 100850, China; hongxiaoxuan1216@163.com (X.H.); hanxiaolu921007@163.com (X.H.); xiaofu0924@163.com (X.L.); lijiale253@163.com (J.L.); 2 School of Pharmacy, Anhui Medical University, Hefei 230032, China

**Keywords:** binder jet 3D printing, compound dispersible tablets, multicompartmental structure, coffee ring, personalized administration

## Abstract

Three-dimensional (3D) printing is an emerging technology that has high application potential for individualized medicines and complex solid dosage forms. This study is designed to explore binder jet 3D printing (BJ-3DP) for the development of high-precision and repeatable compound levetiracetam-pyridoxine hydrochloride (LEV-PN) multicompartmental structure dispersible tablets. PN was dissolved in printing ink directly and accurately jetted into the middle, nested layer of the tablet, and precise control of the drug dose was achieved through the design of printing layers. With modification of the drying method, the “coffee ring” effect caused by drug migration during the curing and molding of the tablets was overcome. Furthermore, 3D topography showed that the tablets have a promising surface morphology. Scanning electron microscopy and porosity results indicated that the tablets have a loose interior and tight exterior, which would ensure good mechanical properties while enabling the tablet to disintegrate quickly in the mouth and achieve rapid release of the two drugs. This study used BJ-3DP technology to prepare personalized multicompartmental structures of drug systems and provides a basis for the development of complex preparations.

## 1. Introduction

Since the 2015 implementation of the Precision Medicine Initiative in the United States, the worldwide demand for personalized and tailored pharmaceutical tablets has risen continually in the past few years. Studies have shown that approximately 80% of adverse effects from drug intake are related to inappropriate doses or combined doses [1]. Thus, maximizing the efficacy and safety of drugs administered to every patient is a priority in modern health care. The need for the development of patient-specific drug dosing has been well recognized, particularly for pediatric and geriatric populations that differ from a “standard patient” in many aspects [2,3]. Therefore, individualized therapy is one of the most efficient strategies. Levetiracetam (LEV) is a second-generation antiepileptic drug that has been widely recognized as the global “gold standard” for treatment of epilepsy because of its minimal adverse effects, good tolerability, and high safety profile. However, there are still side effects that plague patients during long-term use of the drug, including drowsiness, fatigue, dizziness, and behavioral difficulties [4,5], especially for pediatric patients, and the incidence of side effects is higher than that in adults. Pyridoxine hydrochloride (PN) is a water-soluble vitamin that is involved in many important metabolic reactions in the body, such as the transamination of amino acids, regulation of steroid hormone activity, and decarboxylation reactions [6,7]. The lack of PN in children is one of the main causes of epilepsy [8]. Several clinical trials have shown that PN reduced the behavioral side effects associated with LEV [9] and effectively improved the antiepileptic effect of LEV [10,11,12]. However, PN is unstable and decomposes easily under light conditions [13], which causes great difficulties in production and storage.

To improve the efficacy and safety of a therapeutic substance, the pharmaceutical industry has been increasingly pursuing personalized drug delivery systems, and 3D-printing technology has attracted much attention as a novel pharmaceutical manufacturing technique for fabricating patient-tailored medicines [14,15]. Compared with the traditional manufacturing process, 3D-printing technology does not require the development of complicated assembly lines, and the rapid and accurate integrated manufacturing method enables the flexible combination of multiple drug materials, which provides the possibility for complex structural preparations as well as personalized customized production [16,17]. In addition, the use of this technology could reduce exposure to drugs in the production process, minimize the cross-contamination of highly active and difficult-to-clean drugs, and avoid contact with incompatible ingredients during compound preparations, thus solving a series of problems faced with traditional processes [18]. At present, many large pharmaceutical companies are accelerating the development and marketing of 3D printing of tablets.

To date, the only printing process submitted for FDA approval is binder jetting, and the first FDA-approved 3D printed tablet, “Spritam”, is a levetiracetam tablet with oral dispersion properties and a porous internal structure. Unlike traditional tablet manufacturing, the inkjet-based 3D printing process is formed in one piece by layered manufacturing without a compression step, thus providing a porous and rapidly dissolving tablet structure [19,20]. Among 3D-printing techniques, BJ-3DP offers a variety of possibilities for the manufacture of individual and customized preparations [21]. Starting materials (i.e., powders and binder solutions) are already widely used in the pharmaceutical industry, so BJ-3DP can be adapted to manufacturing compared to other 3D-printing technologies [22,23]. It is one of the most promising 3D-printing techniques for the commercial production of pharmaceuticals considered to date [20]. Yu et al. [24] exploited BJ-3DP to develop a zero-order release drug delivery system. Katstra et al. [25] used solutions of Eudragit E-100 in ethanol and RL-PO in acetone as inks to achieve controlled delayed and pulsatile release profiles by combining regions with different solubilities. Syca [26] reported a custom-built binder jet 3D printer for 3D printing pharmaceutical tablets containing the model small-molecule anti-inflammatory drug indomethacin, which has fracture force, friability, and disintegration time comparable to those of commercially available tablets.

The BJ-3DP process could jet a specific dose of active pharmaceutical ingredient (API) solution into a specific area of the tablet. Unlike conventional tablet manufacturing, the drug could be uniformly dissolved or dispersed in the binder and loaded into a specific position in the tablet by jetting. This method of drug delivery makes the process simple and flexible by facilitating control of the internal structure, local composition, and composition differences of tablets, and it also protects the drug dose from fragility or the external environment and enables controlled and personalized administration. However, BJ-3DP still faces some challenges [27], such as clogging of nozzles, drug migration, and bleeding [23,28]. If the API is jetted, the dose level must be precisely controlled [29]. Since BJ-3DP is limited by the thickness of the powder layer, achieving high-resolution objects can also be challenging [30]. Furthermore, the “coffee ring” effect is common in BJ-3DP, and it causes uneven deposition of the printed material and affects the accuracy and performance of the printed product.

In this study, a self-developed binder jet 3D printer was used to develop 3D-printed compound LEV-PN dispersible tablets. The innovation of this study is that the LEV is in the powder mixture, the PN is in the ink, and a precise dose of PN was directly jetted into a specific part of the tablet. The problem of drug photoinstability was solved by partition control, and the precise control of personalized drug dosage was implemented in preparation production. Then, the “coffee ring” effect caused by drug migration during the curing and molding of 3D-printed compound multicompartmental preparations was improved by modifying the drying method. In addition, the microstructure, content, and in vitro release properties of tablets were studied to ensure product quality. This study overcame the technical difficulties faced in preparing 3D printing preparations with complex structures and provides a basis for the development of complex preparations.

## 2. Materials and Methods

### 2.1. Materials

LEV and PN were purchased from Zhejiang Apeloa Jiayuan Pharmaceutical Co., Ltd. (Zhejiang, China) and DSM (Hellen, The Netherlands), respectively. Mannitol (Pearlitol 50C) was purchased from Roquette Frères (Beinheim, France), and microcrystalline cellulose (MCC PH101) was purchased from Asahi Kasei Corporation (Tokyo, Japan). Spearmint flavor and colloidal silica (Aerosil 200) were supplied by Kerry Group (County Kerry, Ireland) and Evonik Degussa GmbH (Marl, Germany), respectively. Sucralose and polyvinylpyrrolidone (PVP) were obtained from Alpha Hi-Tech (Jiangxi, China) and BASF (Ludwigshafen, Germany), respectively. Glycerol was purchased from Nanchang Baiyun Pharmaceutical Co. (Nanchang, China). Sunset yellow pigment and green pigment were purchased from Roha Dyechem Shanghai Co., Ltd. (Shanghai, China). All solvents were of analytical grade.

### 2.2. Preparation of Powder Mixture and Printing Ink

The powder mixture was prepared according to the method in a prior article published by our team [31,32]. Briefly, LEV (API, accounting for 65% of the powder), MCC PH101 (disintegrant), mannitol (filler), PVP (binder), sucralose (sweetener), spearmint flavor (flavoring agent), and Aerosil 200 (glidant) were mixed in a Hopper Mixer (HSD15 Lab Mixer, Canaan Technology, Zhejiang, China) at 20 rpm for 20 min to obtain the final powder mixture.

In this study, 40% (*v*/*v*) isopropanol aqueous solution containing 0.1% (*w*/*w*) PVP, 0.1% (*w*/*w*) green pigment, and 4% (*w*/*w*) glycerin was used as the blank printing ink. Then, 4.5% (*w*/*w*) PN (API) was added to the blank printing ink to obtain the PN printing ink.

### 2.3. Design of the Spatial Structure Model

**Figure 1:**
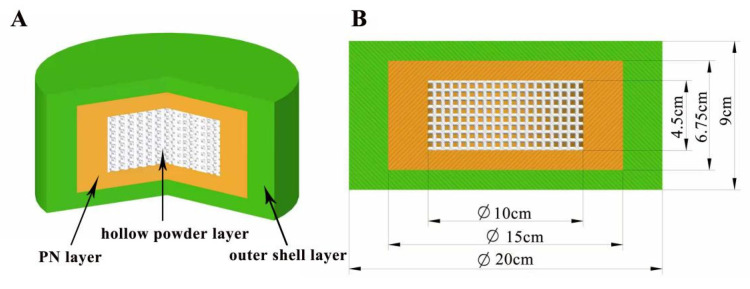
Design diagram of the three-layer nested structure model. (**A**) Three-dimensional section view (from inside to outside: hollow powder layer; PN layer; outer shell layer); (**B**) Dimension drawing of the side view model.

In this study, compound LEV-PN dispersible tablets were designed as a three-layer nested structure. The blank printing ink was jetted onto the outside of the tablet as an outer shell layer, and the PN printing ink was jetted onto selected regions inside the tablet to avoid degradation of the drug by exposure to light during production. The hollow powder layer was formed automatically during the layer printing process without the deposition of printing ink. As shown in Figure 1 below, the hollow powder layer, PN layer, and outer shell layer (without PN) ordered from inside to outside ensured that PN in the nested layer was not exposed to the outside of the tablet. Moreover, the hollow structure was conducive to rapid dispersion of the tablet, which has been confirmed in a study of the model spatial structure of 3D-printed, dispersible LEV tablets [31].

### 2.4. Evaluation of the “Coffee Ring” Effect

For binder jet 3D-printed preparations, API deposition in the printing ink directly affects the accuracy of dosage and the quality of the printed products [33]. It has been found that when the droplet evaporates, it usually deposits a much deeper ring at the edge than in the middle, which is a deposition phenomenon known as the “coffee ring” effect. Deegan et al. [34] first explained the mechanism for formation of the “coffee ring” effect in 1997 and concluded that the phenomenon arose because evaporation at the edge of the droplet was faster than that at the center, resulting in outward capillary flow within the droplet that brings suspended particles to the edge of the droplet and deposits them into a ring.

In this study, the “coffee ring” effect might occur during drying after tablet printing due to the difference in evaporation rates for internal and external parts, resulting in the migration of PN to the tablet surface; this might affect drug distribution and even increase the exposure of PN and affect drug stability. Therefore, regulating the “coffee ring” effect is essential for producing high-precision products via 3D printing.

A stereo fluorescence microscope was used to observe the “coffee ring” effect in the ink drop-printing process. Thus, 0.25% (*w*/*w*) rhodamine (λex = 580 nm, λem = 604 nm) was added to the PN printing ink to serve as a fluorescent marker. After the tablets were printed, they were observed under white and blue light with the stereoscopic fluorescence microscope and photographed at magnifications of 8× and 16×, respectively, and the white and blue light images taken at the same magnification were combined into a single image for comparative examination.

### 2.5. Binder Jet 3D-Printing Process

**Figure 2:**
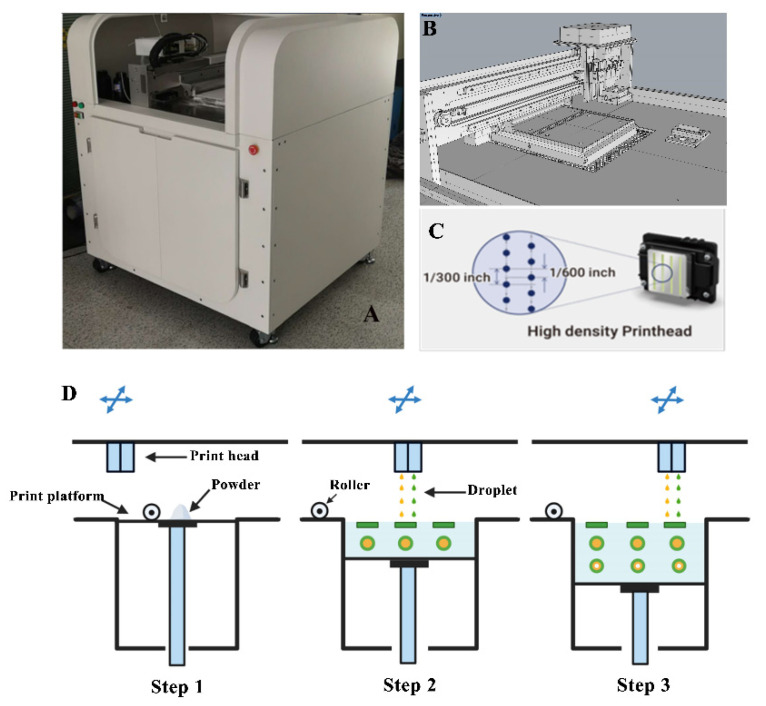
Diagram of the self-developed binder jet 3D printer. (**A**): Physical view of the device; (**B**): Print platform; (**C**): Print head; (**D**): Print process diagram.

This study used self-developed binder jet 3D-printing equipment, as shown in Figure 2. The device was equipped with Epson 4720 piezo printheads with four pairs of eight columns in multipath print heads corresponding to CMYK with 400 nozzles per column, and each print head individually controlled the print ink type and ink drop volume. The device also featured two printing resolutions of 1270 × 600 dots per inch (DPI) and 1270 × 1200 DPI, which provided high-precision and high-quality printing. Firstly, computer-aided design software Geomagic studio and Rhino 6.13 were used to design a model file for the three-layer nested structure. Then, the designed model file was uploaded into the software of the 3D printer, which sliced the model and sent the slices to the 3D printer. The jet volume of the printing ink was set to 2.6 μL/cm^2^, and the resolution was 1270 × 1200 DPI. Two rows of nozzles were set to control the printing of the outer shell layer, and the other two rows of nozzles were set to control the PN layer. The droplet jet mode was set to 5.5 pL. Thin layers of powder mixture were scattered throughout the platform, and the print carriage moved across each layer. The ink solidified the powder only in the cross-section of the designed model, and the remaining powder was used for support. The process was repeated layer by layer until printing was completed. After printing, the tablets were dried to remove organic solvents and excess water, and the supporting powder was recycled for reuse. An air brush was used to remove excess powder and collect the tablets to improve product quality.

### 2.6. Tablet Microstructure

#### 2.6.1. Appearance and Surface Roughness

The surface roughness of the tablets was measured with a three-dimensional white-light interference profilometer (Nexview, ZYGO, Middlefield, CT, USA). The tablets were observed with objective magnifications of 2.75× and 10× and an eyepiece magnification of 1× to evaluate the characteristic surface roughness parameters such as maximum height (Sz), arithmetic mean height (Sa), and root mean square height (Sq), where Sz is the sum of Sp and the maximum valley depth (Sv).

#### 2.6.2. Scanning Electron Microscopy (SEM)

The morphologies of 3D-printed compound LEV-PN multicompartmental dispersible tablets were determined with ultrahigh resolution thermal field emission SEM (JSM-7900F, JEOL, Tokyo, Japan) equipped with an electron control instrument. The conductive layer was sprayed onto the sample surface by using a vacuum evaporator, and then, the accelerating voltage was adjusted to 3.0 kV to observe samples at magnifications of 50× and 300×, respectively.

#### 2.6.3. Porosity

A surface area and pore size analyzer (AutoPore 9520, Micromeritics, Atlanta, GA, USA) was used to determine the porosity of the 3D-printed compound LEV-PN multicompartmental dispersible tablets. The bulk density was measured at 0.22 PSIA, and the apparent density was measured at 29,995 PSIA. The pore size distribution, average pore size, and porosity were recorded. The porosity was calculated as follows:

Porosity (%) = (1 − bulk density/apparent density) × 100%.(1)

### 2.7. Assay and HPLC Analysis

Twenty printed tablets were randomly selected and weighed, finely ground, and then, an appropriate amount (approximately equivalent to 2 mg of PN) was weighed into a 100 mL volumetric flask. The volume of the volumetric flask was made up by adding a mobile phase and sonicated as PN sample solution. The concentrations of LEV and PN were determined using a HPLC system.

The concentration of PN was analyzed by using HPLC (U3000, Thermo, Waltham, MA, USA) with a CAPCELL PAK C18 MGII S5 column (4.6 × 250 mm, 5 μm) maintained at 35 °C. The mobile phase consisted of 15% methanol and 85% (*v*/*v*) sodium pentane sulfonate solution (0.04%, pH 3.0). Elution was performed in isocratic mode with a flow rate of 1.0 mL/min. The injection volume was set at 10 μL, and the signal was detected at a wavelength of 291 nm.

LEV was analyzed using a ZORBOX SB-C8 column (4.6 × 250 mm, 5 μm). The mobile phase was a mixture of acetonitrile and 1.4 g/L anhydrous disodium hydrogen phosphate solution (10:90 *v*/*v*) adjusted to pH 3.5. Samples were eluted in isocratic mode with a flow rate of 1.5 mL/min at 30 °C. The detection wavelength was set at 205 nm, and the injection volume was 20 μL.

### 2.8. In Vitro Drug Release

In vitro drug release of the 3D-printed compound multicompartmental structure dispersible tablets was measured in a USP II paddle apparatus (RC806D, Tianda Tianfa Technology Co., Ltd., Tianjin, China). Six tablets were randomly selected and individually placed in dissolution vessels, each containing 900 mL of phosphate buffer solution (pH 6.8), and the samples were stirred at 50 rpm and 37 ± 0.5 °C. Samples (5.0 mL) were withdrawn at 2.5, 5, 10, 15, 20, and 30 min and filtered through a 0.45 μm filter. Thereafter, 10 μL of the filtered samples was injected into the HPLC system for analysis.

## 3. Results and Discussion

### 3.1. Design of the Spatial Structure Model

**Figure 3:**
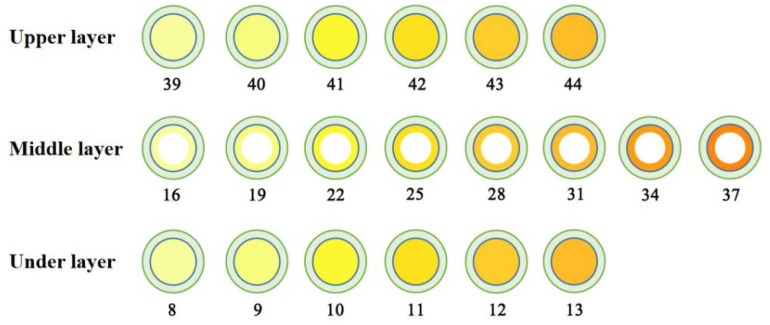
Three-layer nested structure model with PN layer single-layer section diagram.

**Figure 4:**
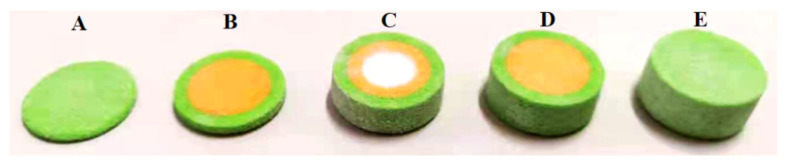
Structure diagram for the 3D-printed compound LEV-PN multicompartmental structured dispersible tablet. (**A**): 1–7 layers; (**B**): 1–13 layers; (**C**): 1–38 layers; (**D**): 1–44 layers; (**E**): 1–50 layers.

The 3D-printed compound dispersible tablets were made of 50 layers, each containing a 180 μm thick layer of powder. Figure 3 shows a single-layer section of the PN-containing layer with a diameter of 20 mm and thickness of 9 mm. The numbers in the diagram represent the number of printing layers (1–7 and 45–50 were the outer shell layers), and there were three regions, the upper, middle and under layers, with a total of 37 layers, among which both the upper and under layers had six layers, and the middle layer had a total of 25 layers. The green area is the outer shell layer, the white area is the hollow layer, and the yellow area is the PN layer; the deeper the yellow area is, the greater the drug content. Figure 4 shows the results of the compound LEV-PN dispersible tablet produced using BJ-3DP. All tablets showed smooth surfaces and regular geometric shapes, and the partitioning between the outer shell layer, PN layer, and hollow layer clearly exhibited high printing accuracy.

**Figure 5:**
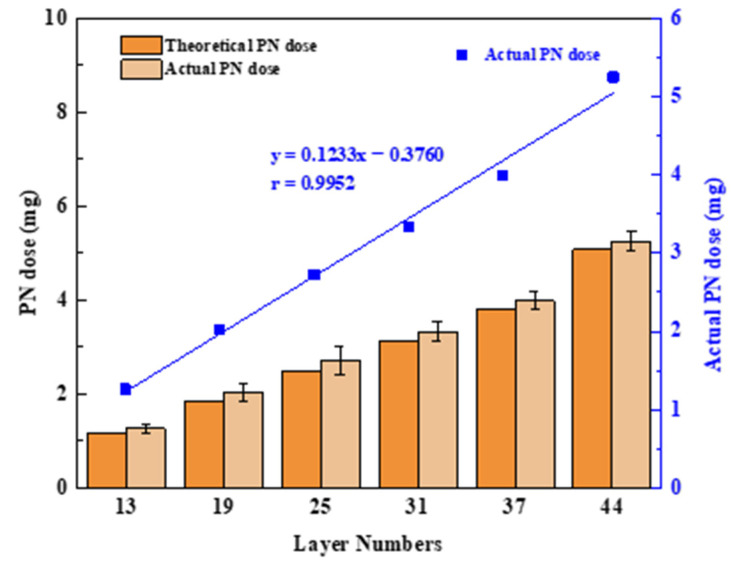
Diagram of the number of printing layers and PN doses; values are expressed as the mean ± SD, *n* = 3.

In this study, the amount of drug loaded in the printing process depended on the jet volume of the PN printing ink. Therefore, the print head must jet continuously and accurately in the printing process, and it has become a great challenge to implement precise and quantitative jetting of ink droplets. This research used software to design six tablet models with different numbers of printing layers, and HPLC was used to measure the PN content and explore the feasibility of the number of printing layers for dose adjustment. Figure 5 shows the good linear correlation between the number of different printing layers (N) and the actual PN dose (C), with an r of 0.9945; this is described by the following formula:

C = 0.1233N − 0.3760 (N ≥ 13).(2)

The product dosage can be adjusted arbitrarily within 1.26–5.25 mg, and the selection of the number of printing layers allowed for small modulations of the drug dose as low as 200 μg. This illustrated that the technology enables precise control of a personalized drug dose by controlling the number of printed layers in the tablet model, which is well suited to fine dose control with highly active drugs or drugs with a narrow therapeutic window. Additionally, it can be seen that the theoretical and actual PN doses are similar at different printing layers. This illustrated the potential of the BJ-3DP to function as a mini-dispenser of tablets with which the dose can be accurately adjusted with an operator’s input into the computer software.

### 3.2. Effect of the “Coffee Ring”

**Figure 6:**
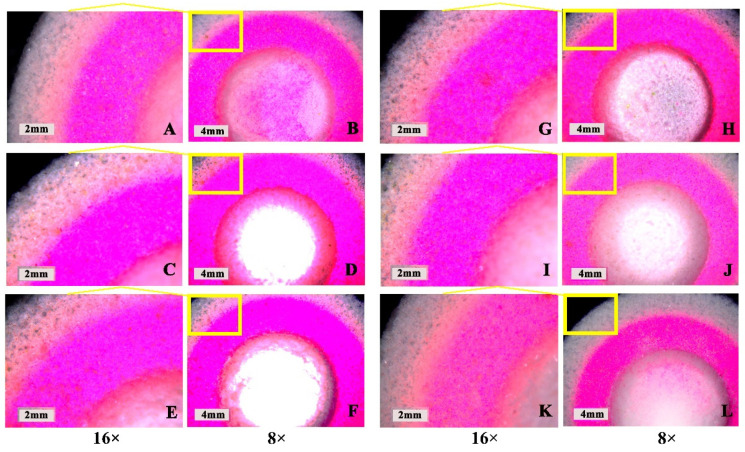
Influence of drying parameters on the “coffee ring” effect of the 3D-printed compound multicompartmental dispersible tablet. (**A**,**B**): Ordinary drying at 30 °C; (**C**,**D**): Ordinary drying at 40 °C; (**E**,**F**): Ordinary drying at 60 °C; (**G**,**H**): Ordinary embedding drying at 40 °C; (**I**,**J**): Vacuum drying at 40 °C; (**K**,**L**): Vacuum embedding drying at 40 °C.

In Figure 6, the pink area is the PN layer (containing fluorescein), and the white–gray area is the outer shell printing layer (without fluorescein). According to the photographed fluorescence shown in Figure 6A–F, fluorescein migrated outwards with different drying temperatures. At 30 °C (Figure 6A,B), the fluorescein in the PN layer of the tablet migrated to the outer layer with the shortest distance. The fluorescein in the PN layer of the tablet migrated farther to the outer shell layer at 40 °C (Figure 6C,D) and 60 °C (Figure 6E,F). This illustrated that the rate for evaporation of the liquid on the outside of the tablet was higher than that on the inside of the tablet and that the outward capillary flow of liquid droplets from the inside of the tablet carried PN to the edge of the tablet and formed a “coffee ring” [35,36] at higher temperatures. However, when the temperature was low, the rate of evaporation of the inside of the tablet was equivalent to that of the outer tablet shell, the outward capillary flow from the inside of the tablet was reduced, and the “coffee ring” phenomenon was significantly alleviated [37]. Therefore, drying temperatures less than 30 °C reduced the “coffee ring” effect to a certain extent. Since the drying efficiency was low at 30 °C, the effect of embedding drying and vacuum drying on the “coffee ring” effect was further investigated by using a drying temperature of 40 °C.

According to the results in Figure 6G–L, the rate of fluorescein migration from the inside to the outside of tablets dried by embedding at 40 °C (Figure 6G,H) and vacuum drying at 40 °C (Figure 6I,J) was significantly slower, indicating that both embedding and vacuum drying could help alleviate the “coffee ring” effect. Embedding drying could reduce the difference in evaporation rate for the PN layer and the outer shell layer of the tablet by increasing the overall drying volume of the tablet, while vacuum drying could accelerate the rate of solvent evaporation inside the tablet under vacuum conditions, thus weakening the influence of the “coffee ring” effect. Moreover, the results of vacuum embedding drying at 40 °C (Figure 6K,L) illustrated that the combination of the two drying methods resulted in better alleviation of the “coffee ring” effect and significant reduction in the migration of fluorescein from the inside to the outside of the tablet; this could avoid the migration of PN to the outside and thus ensure the stability of PN. Therefore, 40 °C vacuum-embedding drying was determined as the drying method for this product.

### 3.3. Tablet Microstructure and Mechanistic Analysis

**Figure 7:**
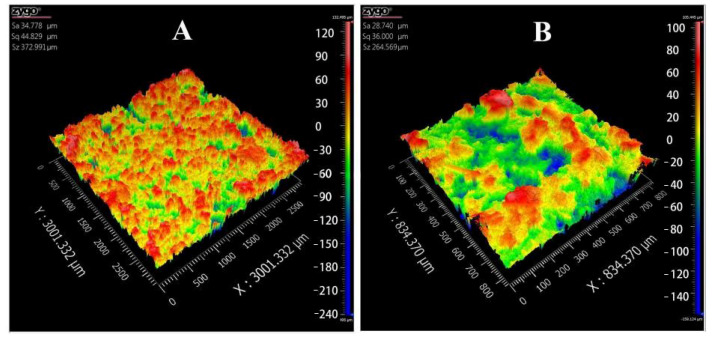
Three-dimensional (3D) topography images of 3D-printed compound dispersive tablets. (**A**): 2.75×; (**B**): 10×.

Poor surface roughness is a common problem in BJ-3DP preparations, and it affects the visual perceptions of patients and may cause dose loss [38,39]. The 3D topography (Figure 7) showed that the 3D-printed compound LEV-PN multicompartmental structure dispersible tablets had a smooth surface. The surface roughness parameters (Table 1) were calculated by using a three-dimensional white light interferometer under 2.75× and 10× objective lenses. Sq and Sa were less than 50 μm, and Sz was less than 400 μm. This indicated that the compound LEV-PN dispersible tablets prepared with self-developed binder jet 3D-printing equipment exhibited a preferable surface morphology and achieved delicate printing.

**Figure 8:**
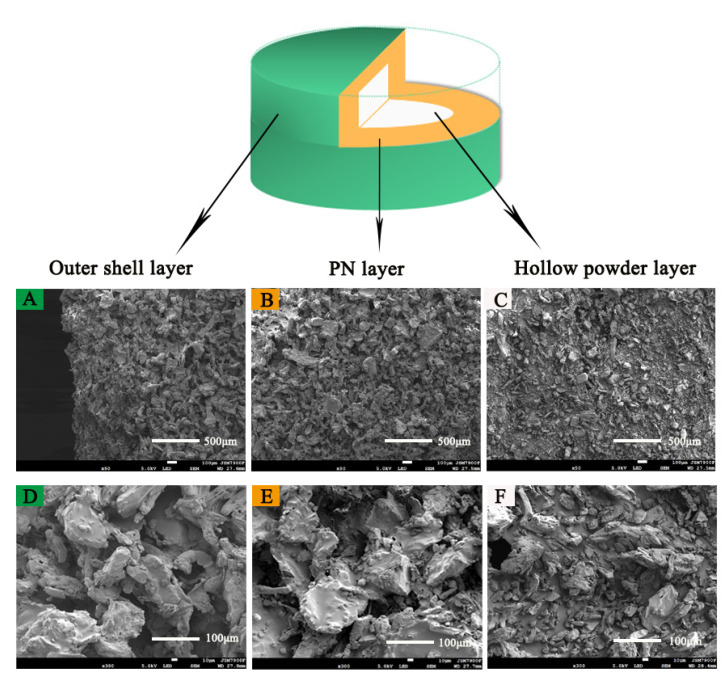
SEM image of the 3D-printed compound multicompartmental structure. (**A**): Outer shell layer (50×); (**B**): PN layer (50×); (**C**): hollow powder layer (50×); (**D**): outer shell layer (300×); (**E**): PN layer (300×); (**F**): hollow powder layer (300×).

Figure 8 shows the microstructures of 3D-printed compound LEV-PN multicompartmental structure dispersible tablets observed by SEM at magnifications of 50× and 300×. The outer shell layer and PN layer of the 3D-printed tablets had similar structural characteristics, which indicated that neither the bonding effect nor spatial structure of the printed area were affected by the addition of PN in the printing ink. It was clear from comparisons that the PN layer and the hollow powder layer of the multicompartmental structure dispersible tablets were different. The PN layer and the outer shell layer were bound well together. Particle sizes were decreased or individual particles could no longer be distinguished as a result of printing of the binder solutions (Figure 8A,B), which could ensure better mechanical properties of the tablet. At the same time, relatively obvious pores can be seen in Figure 8D,E. When the tablets were exposed to water, the water quickly entered the tablet through pore channels, which was conducive to the rapid wetting and dispersion of the tablet. In contrast, the hollow powder layer was uncompacted and showed cracks and fissures among the loose powders, and the particles were almost in their original shapes (Figure 8C,F). The high porosity of the hollow powder layer was certainly beneficial for the penetration of solvent molecules and thus the disintegration of the tablets.

**Figure 9:**
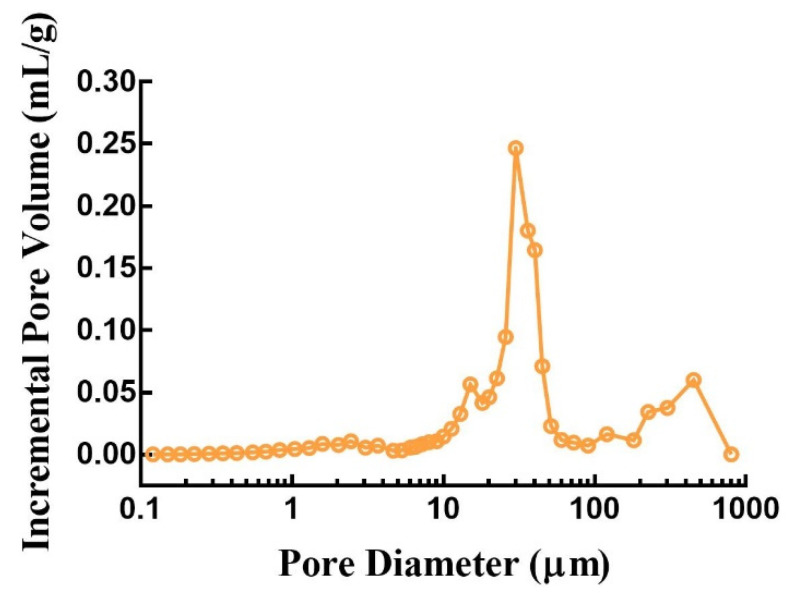
Internal pore size distribution of 3D-printed compound dispersible tablets.

The porosity distribution plot in Figure 9 shows that the 3D-printed compound LEV-PN multicompartmental structure dispersible tablets contained an abundance of internal pores, porosities as high as 63.89%, and an average pore size of 15.65 µm. The high porosity facilitated the rapid penetration of water, which passed through these capillary channels to realize rapid dispersion of the tablets; this was benefited from layered manufacturing and layer-by-layer bonding process characteristics of the 3D-printing preparation and the structural characteristics of the hollow model [31]. However, many 3D-printed products do not have sufficient hardness for post-treatment due to high porosity and poor binding effect among particles [28,40]. In this investigation, the compound LEV-PN multicompartmental structured dispersible tablets exhibited promising mechanical performance, hardness (40.57 ± 1.46 N), and friability (1.8 ± 0.2) (for details, see Table A1 in Appendix A). This might involve two binding mechanisms [41]. On the one hand, the binder contained dissolved PVP, which was left behind when the solvents from the binder liquid evaporated [42]. On the other hand, the mixed powders contained particles of solid PVP, which were activated and acted as a binder upon the absorption of water. Thus, different binding mechanisms were combined to give the compound dispersible tablets sufficient mechanical strength.

### 3.4. In Vitro Drug Release

**Figure 10:**
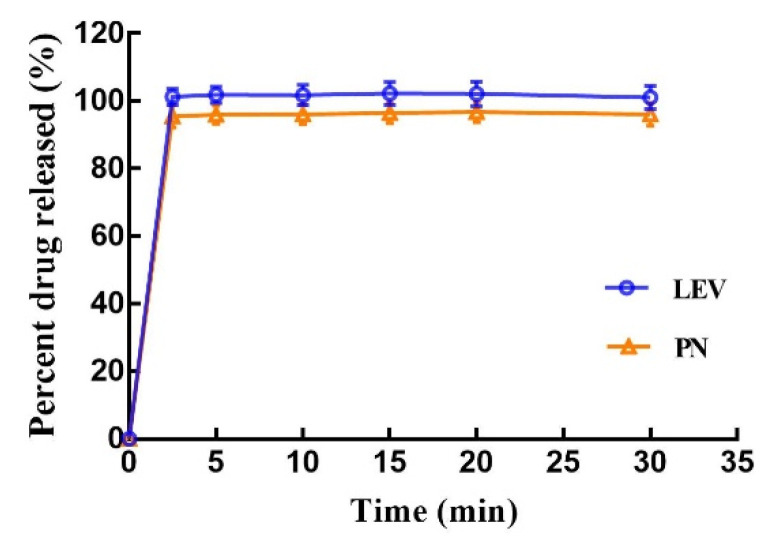
In vitro dissolution profile of the 3D-printed tablets; values are expressed as the mean ± SD, *n* = 6.

Observations made during dissolution showed that the printed tablets were entirely disintegrated at approximately 30 s into the dissolution test. LEV and PN were released from the disintegrated portion of the tablets and dissolved in the dissolution medium. The in vitro drug release profiles in Figure 10 show that both LEV and PN were almost completely released within 5 min. The rapid dissolution or disintegration properties of compound dispersible tablets are closely related to the high wettability and water absorption characteristics of excipients in the tablets. On the other hand, the consolidation behavior of mixed powders in the BJ-3DP process was completely different from those seen with traditional direct compression or granulation methods, which resulted in different mechanisms for rapid disintegration. Conventional tablets tend to rely entirely on the swelling expansion of the disintegrant upon the absorption of water or gas generation by an effervescent disintegrant to achieve sufficient force for rapid disintegration. In contrast, the 3D-printed tablets achieve rapid disintegration due to the following three factors: the high porosity of 3D-printed products, the loose powder in the hollow powder layer, and the use of the hydrophilic polymer PVP as a binder. These results demonstrated that the 3D-printed compound multicompartmental structure dispersible tablets prepared in this research rapidly disintegrated in the mouth and exhibited rapid release, which could be of significant help when administering medications for patients with swallowing difficulties, such as children.

## 4. Conclusions

This study demonstrated that the use of self-developed BJ-3DP allowed the preparation of compound LEV-PN multicompartmental structure dispersible tablets. Based on the photoinstability of PN, a three-layer nested structure was designed. PN was dissolved in the printing ink and directly jetted into the middle, nested layer of the tablet with precision to implement partitioned combination printing, thus avoiding drug stability problems. Additionally, a micro drug system was developed by adjusting the number of printing layers in the model, enabling modulations of drug doses as low as within 200 μg, which is well suited to fine dose control with highly active drugs or drugs with a narrow therapeutic window. Furthermore, drying by vacuum embedding ensured that PN would not migrate to the surface of the tablet, which solved the “coffee ring” effect prevalent in the BJ-3DP process. Microscopic characterizations showed that the preparation had excellent surface morphology and internal structure characteristics, indicating that the ink droplets were accurately jetted during the printing process into specific regions according to the model design, which could achieve fine printing. The loose pore structure enabled the two drugs in the tablet to disintegrate in the mouth quickly and achieve rapid release. This study solved the technical difficulties inherent in preparations for 3D printing multicompartmental structure drug systems and provided a basis for the development of complex preparations.

The innovation of this research lies in the addition of the API to the ink and the development and manufacture of complex multicompartmental structure systems by precisely partitioned jetting with multichannel multinozzles. Firstly, this method of drug delivery does not require mixing the API with the excipients; this reduces the amount of API required for each experiment, which is preferable for material use and reuse. Secondly, the small volumes that can be dispensed by this method of drug delivery, combined with the low concentrations required to prevent nozzle blockage, means that the technology is better suited to printing low therapeutic doses of drugs [29]. Moreover, for low doses of drugs with narrow therapeutic windows, this approach can produce precise, accurate, and reproducible doses and offers potential for the fabrication of doses specific to the patient. However, it is worth noting that addition of the API can change the properties of the ink fluid, which in turn can impede the jetting process. Therefore, the choice of printing ink requires special attention to control viscosity and surface tension [29]. Currently, the equipment we have developed can only be used for precision jetting of low-viscosity printing inks. It is believed that it will be possible to print partitioned combinations of tablets with different drug release behaviors in the future by using print head modulation and printing inks with different viscosities, thereby enabling practical application of the “dosage form design” concept with 3D-printed formulations that meet the needs of patient groups requiring multiple drugs at the same time.

## Figures and Tables

**Table 1:** Surface roughness parameters of 3D-printed compound dispersible tablets.

Parameters	2.75×	10×
Sa	34.778 μm	28.740 μm
Sq	44.829 μm	36.000 μm
Sz	372.991 μm	264.569 μm

## Data Availability

The data presented in this study are available on request from the corresponding author. The data are not publicly available due to some privacy issues about drug development.

## Appendix A

**Table A1:** Weight uniformity, API contents, friability, hardness, disintegration time and dimensions of 3D-printed compound LEV-PN multicompartmental structure dispersible tablets (mean ± SD, *n* = 3).

Drug	Content ± SD (%)	Weight Uniformity (mg)	Friability (%)	Hardness (N)	Dispersion Uniformity (s)	Diameter (mm)	Thickness (mm)
LEV	98.55 ± 2.3	1538.86 ± 1.52	1.8 ± 0.2	40.57 ± 1.46	17 ± 1.5	20.54 ± 0.12	9.43 ± 0.09
PN	100.34 ± 3.5						
